# Positive effect of HPV status on prognostic value of blood lymphocyte-to-monocyte ratio in advanced cervical carcinoma

**DOI:** 10.1186/s12935-016-0334-1

**Published:** 2016-07-04

**Authors:** Si-wei Li, Wenxin Yuan, Bo Zhao, Zhuo-kai He, Xiang Guo, Wei-xiong Xia, Li-hua Xu

**Affiliations:** Department of Radiation Oncology, the Affiliated Hospital of Guilin Medical University, Guilin, 541004 People’s Republic of China; Department of Ultrasonography, the First Affiliated Hospital, Nanchang University, Nanchang, People’s Republic of China; Department of Nasopharyngeal Carcinoma, State Key Laboratory of Oncology in Southern China, Guangzhou, 510060 People’s Republic of China; Department of Hematology, the First Affiliated Hospital of Guangzhou Medical University, Guangzhou, 510230 People’s Republic of China; Guangdong Key Laboratory of Urology, the First Affiliated Hospital of Guangzhou Medical University, Guangzhou, Guangdong People’s Republic of China

**Keywords:** Cervical carcinoma, Human papilloma virus, Prognosis, Lymphocyte to monocyte ratio, Hybrid capture 2

## Abstract

**Background:**

This retrospective study aimed to investigate the prognostic significance of pretreatment lymphocyte-to-monocyte ratio (LMR) in locally advanced cervical cancer and its effect on overall survival.

**Methods:**

The usual blood routine test was quantitatively performed to detect LMR. Signal strengths of human papilloma virus (HPV) type DNA in detected cervical cancer samples using hybrid capture 2 were analyzed in relative light units (RLU) compared with 1 pg/mL of HPV type 16 DNA-positive control (RLU/PC) samples. A total of 1.0 RLU/PC (~1 pg/mL) was used as the threshold for a positive result. The HPV-positive specimens were typed using reverse-hybridization line probe assay.

**Results:**

The LMR and HPV DNA were found to be independent prognostic markers for 5-year overall survival (OS) and progression-free survival, respectively. Their joint detection may further enhance the predictive value for OS. In the positive HR (high risk)-HPV DNA status subgroup, LMR had a positive effect on improved OS but not in the non-HR HPV DNA status subgroup.

**Conclusions:**

The LMR and HR-HPV DNA status can be identified as independent prognostic factors. The different influences of LMR in combined chemoradiotherapy on survival may be related to HR-HPV DNA status. The combined detection of LMR and HR-HPV DNA status may contribute to screening prognosis.

## Background

Cervical cancer is the third most commonly diagnosed type of cancer and the fourth leading cause of cancer death in women, accounting for 8 % (275, 100) of total cancer deaths among female individuals in 2008 [1]. Chemoradiotherapy is considered as a standard treatment option for patients with unresectable and locally advanced cervical carcinoma. The 5-year survival rate of advanced cervical carcinoma has been significantly improved owing to the application of concurrent chemoradiotherapy in recent years. However, local recurrence and distant metastasis are still the most common failure patterns for advanced cervical cancer. Once post-treatment failure occurs, patients have weaker prognosis with 1-year survival rate of less than 20 % [2]. Despite this alarming fact, more efficient methods to predict prognosis are still lacking. Therefore, a reliable and effective method must be explored to screen patients at high risk (HR) of poor prognosis, thus providing reasonable basement for more tailored cancer therapy.

Inflammation has long been associated with the development, progressive process, and long-time treatment outcomes of cancers. Pretreatment peripheral differential leukocytes (including neutrophil, lymphocyte, and monocyte) are associated with prognosis of various malignancies, such as breast cancer [3], gastric cancer [4], acute lymphoblastic leukemia [5], lymphoma [6, 7], primary hepatocellular carcinoma [8], and nasopharyngeal carcinoma [9].

To date, only one study has examined the relationship between differential leukocyte levels and prognosis of patients with cervical cancer, but this study only investigated the prognostic value of the neutrophil-to-lymphocyte ratio (NLR) in cervical cancer [10]. The prognostic significance of lymphocyte-to-monocyte ratio (LMR), which is calculated by dividing absolute lymphocyte count (ALC) by absolute monocyte count (AMC), in cervical cancer has not been clarified. Pretreatment LMR has been reported to be a prognostic factor for clinical outcomes in non-cervical cancers, such as diffuse large B cell lymphoma, Hodgkin’s lymphoma, and nasopharyngeal carcinoma [6, 7, 9]. Therefore, we hypothesize that lymphocytes, monocytes, and LMR may also play an important role in cervical carcinoma. Previous studies have demonstrated that HPV DNA status is linked to prognosis in invasive cervical cancer [11]. However, whether the HPV DNA status has some influence on the prognosis of LMR level in cervical cancer remains unclear.

Therefore, we performed a retrospective study on the prognostic value of pretreatment LMR in locally advanced cervical carcinoma patients and then investigated its correlation with HPV DNA status and the influence of HPV DNA status on the prognosis of LMR level in the disease. To the best of our knowledge, this is the first study on the association between LMR and prognosis of cervical carcinoma patients.

## Patients and methods

### Patients and general data

Patients with locally advanced cervical carcinoma, who were initially treated at the Sun Yat-sen University Cancer Center from January 2003 to December 2004, were enrolled in this study. Patient eligibility was based on the following inclusion criteria before treatment: (1) biopsy-confirmed cervical cancer of clinical stage IIb-IVa according to the International Federation of Gynecology and Obstetrics (FIGO) 2009 Staging System; (2) histological types that were squamous cell carcinoma (SCC) or adenocarcinoma (ADC) or their mixture in cervical carcinoma; (3) definitive radiotherapy combined with chemotherapy in any sequence; (4) with complete clinical data; (5) performance status ranging from 0 to 2 based on the Eastern Cooperative Oncology Group system; (6) normal renal, cardiac and liver functions; and (7) with sufficient pretreatment tumor biopsy specimens for measurement of HPV DNA levels before treatment.

The exclusion criteria were as follows: (1) distant metastasis under clinical examination or imaging at the time of diagnosis; (2) histological types that were not squamous cell carcinoma (SCC) or adenocarcinoma (ADC) or their mixture in cervical carcinoma; (3) previous chemotherapy; (4) previous radiotherapy to the pelvis region; (5) previous surgery on the primary tumor site before being enrolled in this study; (6) history of other malignant tumors or simultaneous multiple tumors; and (7) a positive pregnancy test result for women of childbearing age.

A total of 424 patients were enrolled in this study. Patient consent and approval from the Institute Research Ethics Committee were obtained prior to the use of these clinical materials for research purposes. The clinical characteristics of the locally advanced cervical carcinoma patients are described in detail in Table 1. The enrolled patients had a median age of 47 years (ranging from 18 to 74 years). Clinical follow-up information was obtained from patient medical records.

**Table 1:** Correlation of pretreatment LMR with clinicopathologic characteristics of patients with advanced cervical carcinoma (*n* = 424)

Characteristic	No. of patients (%)	Pretreatment LMR level		*p* value	HPV DNA status		*p* value
		High value (*n* = 260)	Low value (*n* = 164)		HR-positive (*n* = 390)	Non-HR (*n* = 34)	
*Age, years*							
≤46	205 (48.35 %)	133	72	0.146	190	15	0.607
>46	219 (51.65 %)	127	92		200	19	
*HPV DNA*							
Positive	390 (91.98 %)	246	144	0.012	–	–	–
Negative	34 (8.02 %)	14	20		–	–	
*Lymph node status classification*							
Node-negative	277 (65.33 %)	212	65	0.001	252	25	0.295
Node-positive	147 (34.67 %)	48	99		138	9	
*FIGO classification*							
IIA	86 (20.28 %)	41	45	0.0 22	81	5	0.803
IIB	84 (19. 81 %)	48	36		78	6	
IIIA	92 (21.70 %)	63	29		83	9	
IIIB	90 (21.23 %)	61	29		81	9	
IVA	72 (16.98 %)	47	25		67	5	
*Pathological type*							
SCC	305 (71.93 %)	194	111	0.337	288	17	0.016
ADC	31 (7.31 %)	19	12		25	6	
ASC	33 (7.78 %)	19	14		29	4	
UDC	55 (12.97 %)	28	27		48	7	
*Treatment modality*							
IC + RT	143 (33.73 %)	83	60	0.607	132	11	0.984
CCRT	147 (34.67 %)	92	55		135	12	
IC + CCRT	134 (31.60 %)	85	49		123	11	

*LMR* lymphocyte-to-monocyte ratio; *HPV* human papillomavirus; *DNA* deoxyribonucleic acid; *SCC* squamous cell carcinoma; *Ag* antigen; *FIGO* International Federation of Gynecology and Obstetrics; *ADC* adenocarcinoma; *ASC* adenosquamous carcinoma; *IC* inductive chemotherapy; *CCRT* concurrent chemoradiotherapy; *RT* radiation therapy; *HR* high risk

All enrolled patients received any of the three treatment modalities: inductive chemotherapy plus radiation therapy (IC + RT), concurrent chemoradiotherapy (CCRT), and inductive chemotherapy (IC) plus CCRT.

## Treatment

### Radiation

Images taken from computed tomography (CT) with immobilization tools were used to plan external beam radiation (EBRT) and brachytherapy. Each patient received EBRT using 6 MV photons with a 7-field intensity modulated radiotherapy (IMRT) technique to spare some of the small bowels anterior to the iliac nodes. EBRT was administered to the whole pelvis for a total dose of 50 Gy. The daily fraction size was 2.0 Gy, which was administered five times per week for 5 weeks. High-dose rate intracavitary brachytherapy was started 3–4 weeks after EBRT initiation. The median dose of 24 Gy was delivered to point A with 4 Gy per fraction twice a week for 3 weeks. The total dose delivered to point A was equal to or greater than 85 Gy.

#### Chemotherapy

IC was administered 3 weeks per cycle for two or three cycles, with a combination of cisplatin (80 mg/m^2^) and paclitaxol (175 mg/m^2^). All concurrent chemotherapy regimens were cisplatin-based. Cisplatin (50 mg/m^2^) alone or a combination of cisplatin (30 mg/m^2^) and paclitaxol (135 mg/m^2^) was administered weekly for 5 weeks during EBRT, beginning on the first day of radiation.

### LMR measurement

Peripheral blood samples were obtained from the peripheral blood of patients after cervical cancer diagnosis and before the initiation of any treatment modality. Peripheral lymphocytes and monocytes were counted using the automated hematology analyzer Sysmex XE-2100 (Sysmex, Japan). All patients had no self-reported acute infections or hematologic disorders, indicating that the cell counts can represent the normal baseline value. The peripheral LMR was calculated from the differential count by dividing ALC by AMC.

### Detection of HPV DNA status

Paraffin wax-embedded blocks from 424 patients with locally advanced cervical cancer were collected. Deparaffinization was performed with Xylene to prepare samples for DNA isolation, and DNA was retrieved using the Qiagen DNeasy Blood & Tissue Kit (Valencia, CA, USA). DNA was extracted using proteinase K digestion and then purified by a modified protocol using the Qiagen DNeasy Kit, as described by Steinau [12]. The extracted DNA was amplified using the PCR-SPF 10 (version 1) method. The signal strengths of HPV DNA in cervical cancer samples were detected using Hybrid Capture 2 (HC2), a DNA enzyme immunoassay, and were analyzed in relative light units (RLU) compared with 1 pg/mL of HPV-type 16 DNA-positive control (RLU/PC) samples. A total of 1.0 RLU/PC (~1 pg/mL) was used as the threshold for a positive result.

### Genotyping of positive HPV DNA

The HPV-positive specimens were typed using reverse-hybridization line probe assay with 23 type-specific hybridization probes [13, 14]. We defined oncogenic HPV 16, 18, 31, 33, 35, 39, 45, 51, 52, 56, 58, 59, 66, and 68 as HR HPV type and HPV 6, 11, 42, 43, 81, and 83 as low-risk (LR) HPV type [15, 16]. The possible HR and LR HPV types were reclassified as non-HR HPV types.

### Follow-up and statistical analysis

Follow-up examination was performed approximately every 3 months for the first 2 years, every 6 months for the next 3 years, and every year thereafter. During the routine follow-up, imaging studies, including pelvic magnetic resonance imaging, thorax and abdomen CT, and bone scan using single photon emission CT, were performed at the predetermined time. When tumor recurrence and/or distant metastasis was suspected based on clinical findings or imaging studies, biopsy of that lesion was performed on a case-by-case basis.

The observations ended on December 31, 2012. The assessed endpoints of this study were overall survival (OS) and progression-free survival (PFS). OS was characterized by the time of the initial treatment to death from cervical carcinoma or the last follow-up visit. PFS was characterized by the last date of follow-up or the end date of observation from the initial treatment to distant metastasis or the relapse noted on images in patients who completely responded to treatment, and the locoregional or distant progression of disease in patients who partially responded.

These endpoints were analyzed and compared using the Kaplan–Meier method and log-rank test. Chi square test was used to compare qualitative variables. Univariate analysis was performed to determine the significance of variables using the Cox regression model for PFS and OS. Multivariate analyses with the Cox proportional hazards regression model was used to determine the independent prognostic factors. Spearman’s rank correlation test was used to evaluate the relationship between LMR level and HPV DNA status in terms of some clinical characteristics. The statistical test was a two-sided test performed using SPSS 16.0 programs (SPSS Inc., Chicago, USA). A two-tailed *p* value <0.05 was considered significant.

### Classification of the enrolled patients according to the detected HPV DNA status from pretreatment cancer tissue

According to the detected HPV DNA status from the pretreatment cancer tissue of patients with advanced cervical carcinoma, the patients with positive HR HPV DNA were classified as the HR-positive HPV DNA group, whereas those with negative or low risk-HPV DNA were classified as the non-HR HPV group.

### Selection of appropriate cut-off scores for PFS and OS

Receiver operating characteristic curve analysis was used to define the most appropriate cutoff value for absolute lymphocyte and monocyte counts and LMR to stratify patients at HR of malignancy-related death or progression, thereby avoiding the predetermined cut point. The score selected as the best cut-off value was the one closest to the point with maximum sensitivity and specificity.

## Results

### Follow-up

In this study involving 424 patients with unresectable advanced cervical carcinoma treated with chemoradiotherapy, none had distant metastasis at the time of diagnosis. The median follow-up period was 73 months for all patients and 88 months for patients who did not die of their disease. A total of 129 patients (30.43 %, 129/424) had distant metastasis throughout the follow-up period, of which 52, 40, 25, and 12 developed bone metastases, lung metastases, liver metastases and distant lymph node metastases in the abdominal or thoracic cavity, respectively. Moreover, 80 patients developed recurrent disease at either local or regional lesion site; the local–regional recurrence rate was 18.87 % (80/424). The 3- and 5-year OS rates were 70.3 and 54.76 %, respectively. Of 148 patients, 52 died from metastatic disease, 54 from recurrent and metastatic disease, and 42 from recurrent disease.

**Fig. 1:**
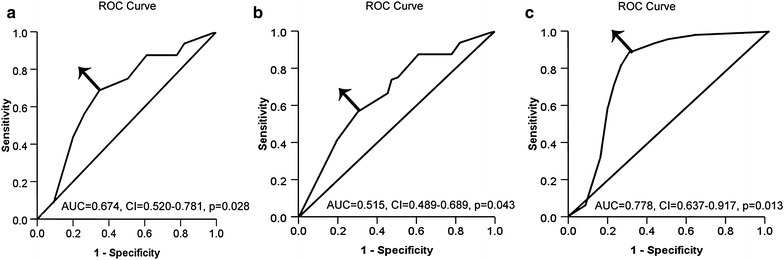
ROC curve analysis to assess the optimal cutoff value of each pretreatment peripheral blood cell in patients with unresectable advanced cervical carcinoma treated with chemoradiotherapy. **a** ROC curves analysis for ALC at diagnosis; **b** ROC curves analysis for AMC at diagnosis; **c** ROC curves analysis for LMR at diagnosis. *Arrow* indicates the pertinent point on the curve that was identified as the better balance between sensitivity and specificity. *ROC* receiver operating characteristic; *AMC* absolute monocyte count; *ALC* absolute lymphocyte count; *LMR* lymphocyte-to-monocyte ratio

A total of 424 patients were eligible for this study. The optimal cut-off values of ALC, AMC, and LMR were 2.35 × 10^9^/L (AUC = 0.674,** 95 %** CI 0.520–0.781, *p* = 0.028) with sensitivity of 77.73 % and specificity of 72.4 %, 0.38 × 10^9^/L (AUC = 0.515,** 95 %** CI 0.489–0.689, *p* = 0.043), and 5.28 (AUC = 0.778,** 95 %** CI 0.637–0.917, *p* = 0.013) with sensitivity of 85.37 % and specificity of 75.40 %, respectively (Fig. 1a, b, c). Patients with HR-positive HPV DNA exhibited higher monocyte counts than those with non-HR HPV DNA(*p* < 0.01).

### Correlation of LMR Level or HPV DNA status with clinical characteristics and their mutual relationship

Of the 424 patients with advanced cervical carcinoma, 390 had HR-positive HPV DNA in the cancer tissue samples tested using HC2. As shown in Tables 1 and 2, no significant difference in the case of different ages and treatment modalities were found for pretreatment LMR Level and HPV DNA status, respectively. Moreover, the LMR level was not significantly different (*p* > 0.05) compared with the WHO pathologic type. However, the LMR level was significantly different between different lymph node status classifications and FIGO classification (*p* < 0.05), as summarized in Table 1. Contrary to LMR level, the HPV DNA status showed no significant relationship with lymph node status and FIGO classifications except with the WHO pathologic type (*p* < 0.01).

**Table 2:** Univariate analysis of variables associated with 5-year OS and PFS (n = 424)

Prognostic factors	OS			PFS		
	Hazard ratio	95 % CI	*p* value	Hazard ratio	95 % CI	*p* value
LMR: high versus low	0.528	0.129–0.849	0.008	0.541	0.136–0.894	0.002
HPV DNA status: HR-positive HPV DNA versus non-HR HPV DNA	0.404	0.025–0.647	0.001	0.529	0.151–0.826	0.001
Age : ≤46 versus >46	0.452	0.121–0.837	0.014	0.412	0.087–1.030	0.040
FIGO classification: IIA versus IIB versus IIIA versus IIIB versus IIIB versus IVA	0.197	0.018–0.416	0.034	0.484	0.0134–0.839	0.024
pathological type: SCC versus ADC versus ASC versus UDC	0.418	0.168–0.827	0.031	0.478	0.174–0.930	0.043
Lymph node status classification: positive lymph node versus negative lymph node	3.586	0.876–5.439	0.043	2.542	1.047–4.283	0.045
Treatment modality: IC + RT versus CCRT versus IC + CCRT	0.530	0.129–0.934	0.039	2.164	1.687–4.017	0.102
ALC: high versus low	0.571	0.292–0.843	0.002	0.513	0.385–0.738	0.008
AMC: high versus low	2.418	1.215–4.738	0.931	0.714	0.306–1.136	0.035

*OS* overall survival; *PFS* progression-free survival; *CI* confidence interval; *LMR* lymphocyte-to-monocyte ratio; *HPV* human papillomavirus; *DNA* deoxyribonucleic acid; *HR* high risk; *FIGO* International Federation of Gynecology and Obstetrics; *SCC* squamous cell carcinoma; *ADC* adenocarcinoma; *ASC* adenosquamous carcinoma, *UDC* undifferentiated carcinoma; *IC* inductive chemotherapy; *CCRT* concurrent chemoradiotherapy; *RT* radiation therapy; *AMC* absolute monocyte count; *ALC* absolute lymphocyte count

In this study, the LMR levels were closely associated with HPV DNA status. Most of the patients with high LMR levels had HR-positive HPV DNA. The significant association between high LMR level and HR-positive HPV DNA rate was observed not only in terms of FIGO classifications II_A_ (r = 0.421, *p* = 0.000), II_B_ (r = 0.397, *p* = 0.001), III_A_ (r = 0.571, *p* = 0.000) and III_B_ + IV_A_ (r = 0.720, *p* = 0.000), but also on OS (r = 0.361, *p* = 0.000) and PFS (r = 0.352, *p* = 0.026).

### Prognostic implications of LMR level and HPV DNA status from pretreatment cancer tissue

**Fig. 2:**
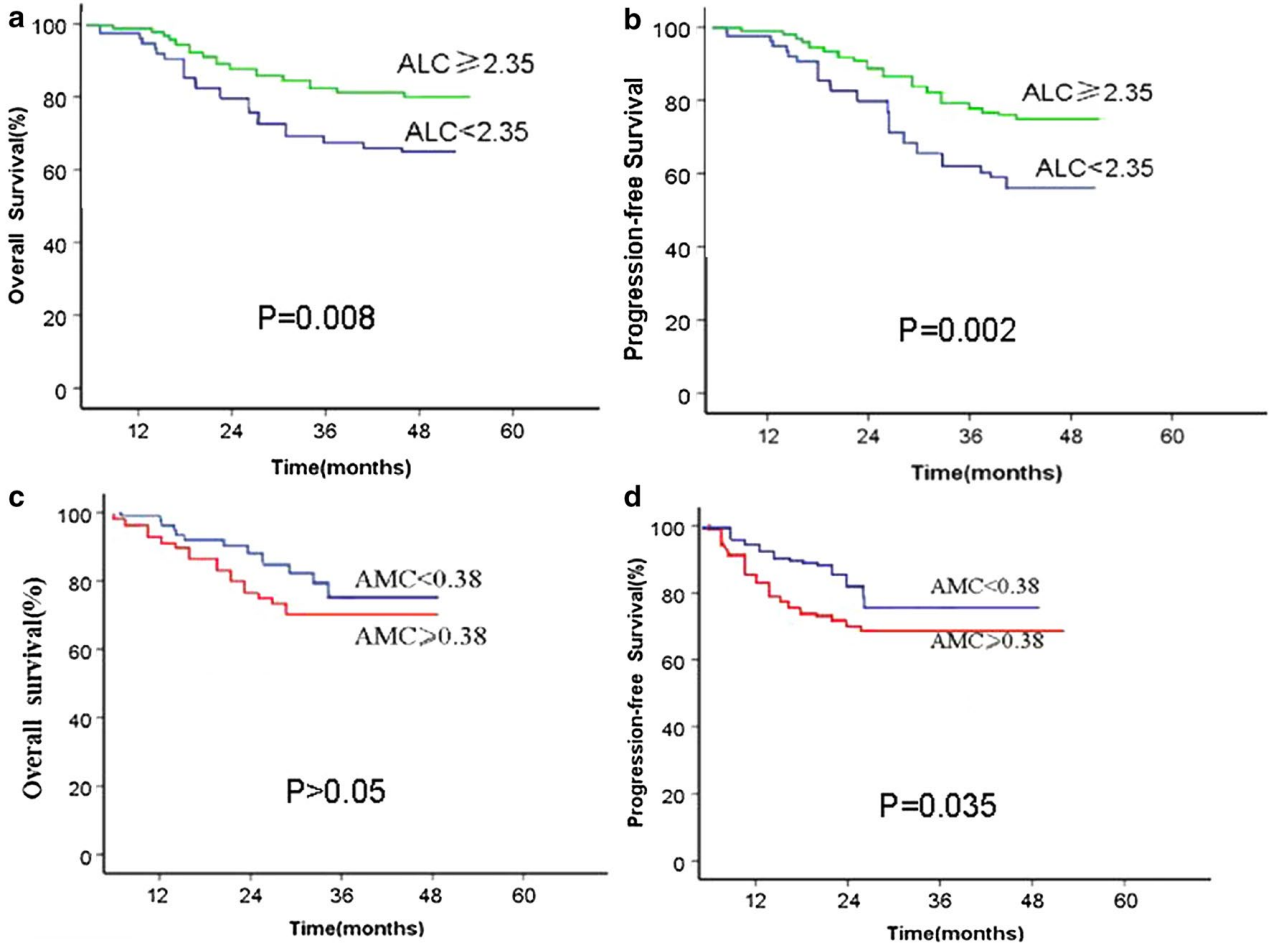
Kaplan–Meier survival curve for patients with unresectable advanced cervical carcinoma patients according to each pretreatment peripheral blood cell. **a** ALC and OS; **b** ALC and PFS; **c** AMC and OS; and **d** AMC and PFS

The 5-year OS and PFS of patients in the high ALC (≥2.35 × 10^9^) and low ALC (<2.35 × 10^9^) groups were analyzed and found to be 82.2 and 64.2 % (*p* = 0.008, Fig. 2a) and 77.2 and 56.9 % (*p* = 0.002, Fig. 2b), respectively.

A significant difference was observed between the high AMC (≥0.38 × 10^9^) and low AMC (<0.38 × 10^9^) groups on 5-year PFS, with 79.5 and 71.8 %, respectively (*p* = 0.035, Fig. 2d). However, no significant difference regarding the 5-year OS was found between the high AMC (≥0.38 × 10^9^) and low AMC (<0.38 × 10^9^) groups, with 75.5 and 71.5 %, respectively (*p* > 0.05, Fig. 2c).

**Fig. 3:**
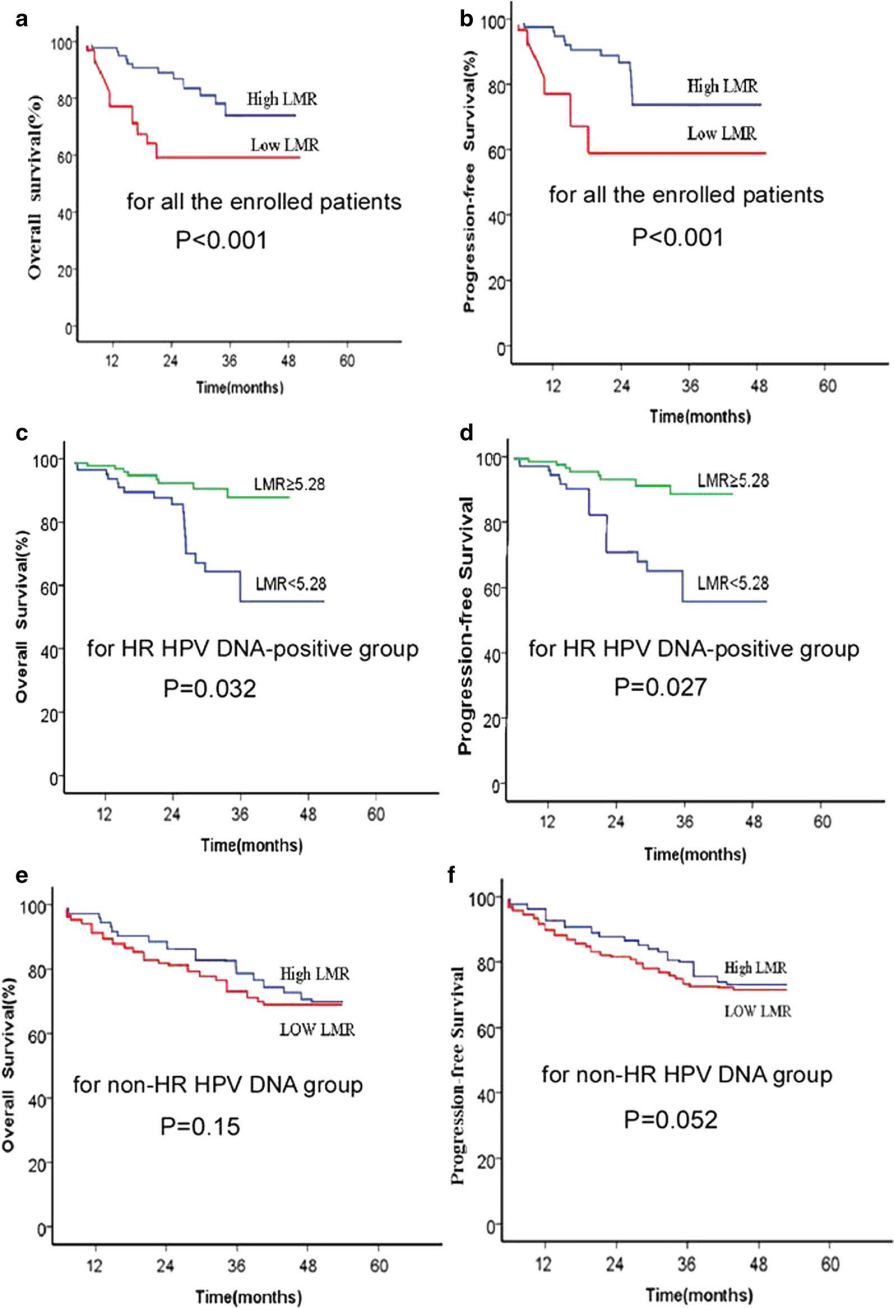
Kaplan–Meier survival curve for patients with unresectable advanced cervical carcinoma patients according to LMR. **a** All the enrolled patients and OS; **b** All the enrolled patients and PFS; **c** HR-positive HPV DNA group and OS; **d** HR-positive HPV DNA group and PFS; **e** non-HR HPV DNA group and OS; and **f** non-HR HPV DNA group and PFS

The 5-year OS rates of the high LMR group (≥5.28) and low LMR group (<5.28) were analyzed using the he Kaplan–Meier survival analysis and were found to be 75.0 and 61.5 %, respectively. The difference between the two groups was significant in terms of the log-rank test (*p* = 0.000, Fig. 3a). Furthermore, patients in the high LMR group had longer PFS than those in the low LMR group (*p* = 0.000, Fig. 3b).

In the HR-positive HPV DNA group, the 5-year OS and PFS rates were 82.5 versus 54.8 % (*p* = 0.032) and 83.9 versus 50.9 % (*p* = 0.027) for the high LMR group versus the low LMR group before treatment (Fig. 3c, d), respectively. However, for the non-HR HPV DNA group, no significant difference in the 5-year OS was found between the two groups, with 66.8 and 65.9 % (*p* > 0.05) (Fig. 3e). A tendency of significant difference existed on PFS, with 72.8 and 57.8 % (*p* = 0.052, Fig. 3f).

**Fig. 4:**
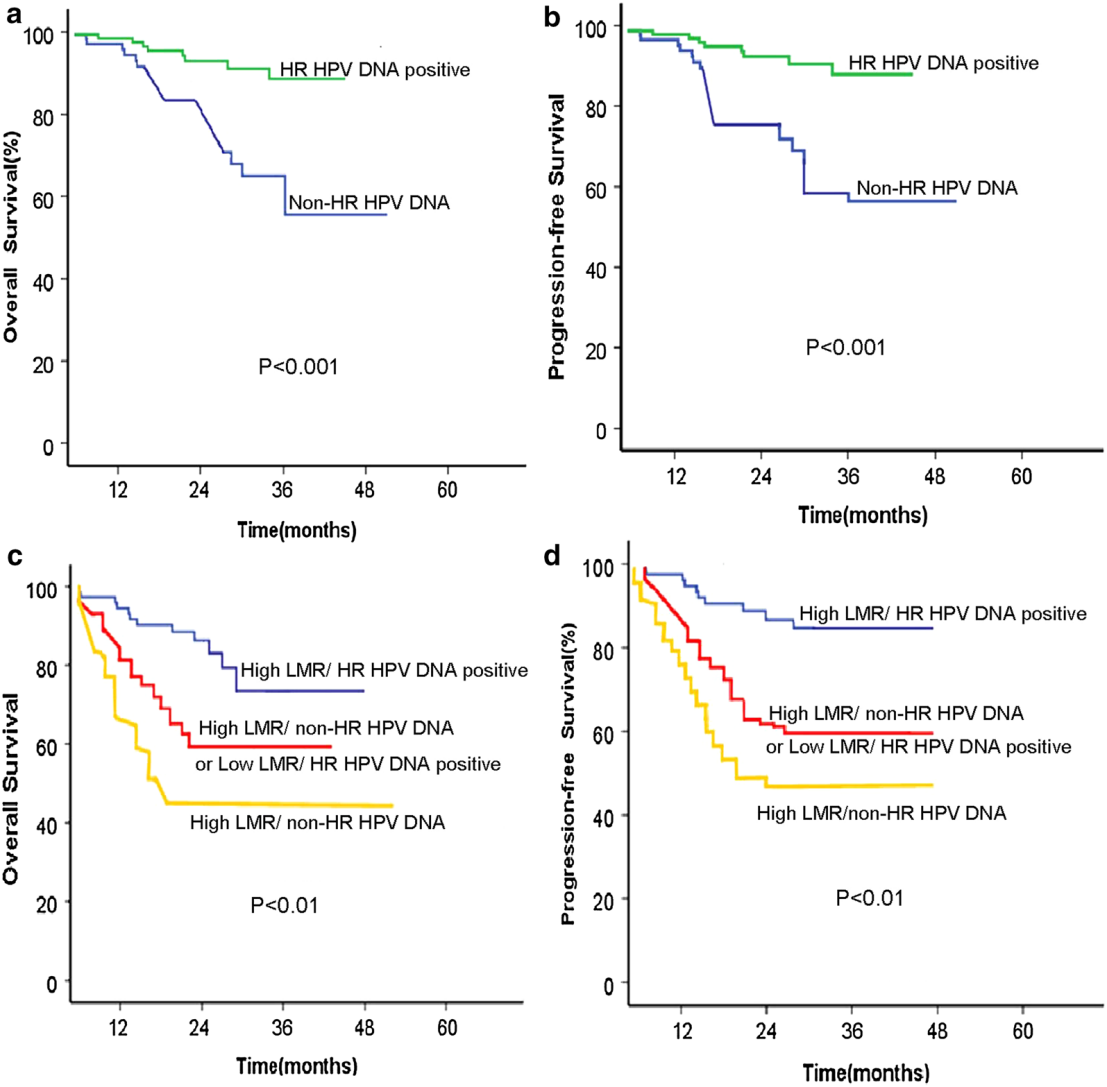
Kaplan–Meier survival curve for patients with advanced cervical carcinoma according to HPV DNA status or combined modality based on pretreatment LMR level and concurrent HPV DNA status. **a** HPV DNA status and OS; **b** HPV DNA status and PFS; **c** combined modality and OS; and **d** combined modality and PFS

The 5-year OS and PFS rates were 81.7 versus 58.4 % (*p* = 0.030) and 82.5 versus 52.0 % (*p* = 0.032) for the HR-positive HPV DNA group versus the non-HR HPV DNA group before treatment (Fig. 4a, b), respectively.

### Prognostic implications of LMR level and concurrent HPV DNA status from pretreatment cancer tissue

Each combined expression of LMR level and concurrent HPV DNA status in a single patient before treatment formed three different combined groups, namely, high LMR/HR-positive HPV DNA, high LMR/non-HR HPV DNA or low LMR/HR-positive HPV DNA, and low LMR/non-HR HPV DNA group. As shown in Fig. 4c and d, patients with high LMR and concurrent HPV-positive DNA showed the best prognosis because of their longer OS times than any other groups on OS and PFS (*p* < 0.01, both). This group was followed by patients with high LMR/non-HR HPV DNA or low LMR/HR-positive HPV DNA, and finally by those with low LMR/non-HR HPV DNA.

### Univariate analysis of variables associated with 5-year OS and PFS

**Fig. 5:**
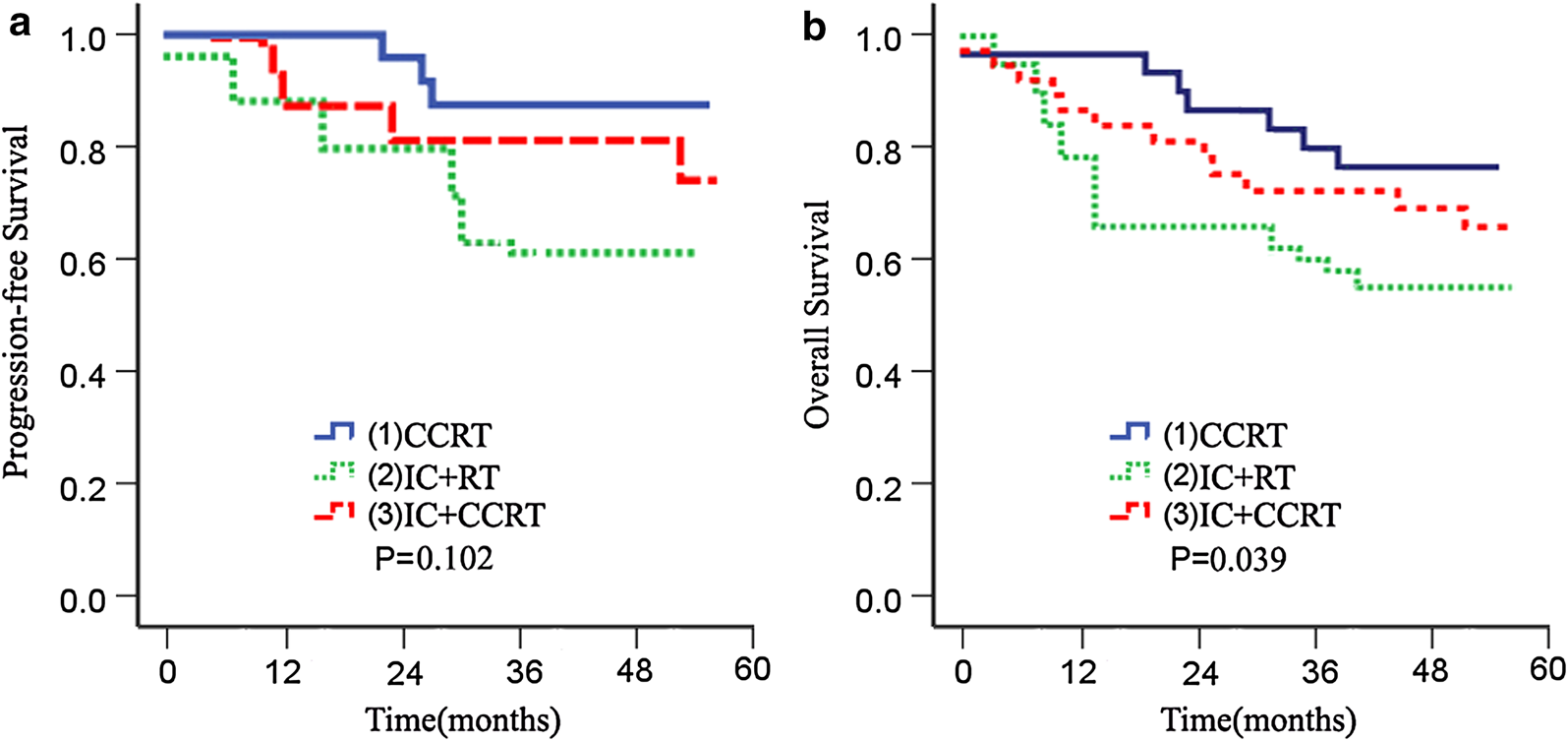
Kaplan–Meier survival curve for patients with advanced cervical carcinoma according to treatment modalities. **a** Treatment modalities and PFS; **b** Treatment modalities and OS

In univariate analysis, we revealed that most of clinopathological factors (LMR level, HPV DNA status, age, FIGO classification, lymph node status classification, and ALC) were related to OS, and PFS, respectively, as summarized in Table 2. AMC was not significantly related to OS, although it was significantly related to PFS. Contrary to AMC, treatment modalities were significantly related to OS (HR = 0.530, 95 % CI 0.129–0.934, *p* = 0.039), although they were not significantly related to PFS (*p* = 0.102, Fig. 5).

### Multivariate Cox risk model analysis

Some variables proved significant in the univariate analysis, were further introduced into the Cox regression model using the Enter method, revealing that pathological type and HPV DNA status were independent predictors of OS, and PFS, respectively, in addition to FIGO classification (HR = 3.486, 95 % CI 0.987–5.439, *p* = 0.035) of OS, for all the enrolled patients (Table 3). Treatment modalities were independent predictors of OS, but not of PFS, whereas ALC were independent predictors of OS, but not of PFS (Table 3).

**Table 3:** Multivariate analysis of Cox proportional hazards model 5-year of OS and PFS for all the enrolled patients (n = 424)

Prognostic factors	OS			PFS		
	Hazard ratio	95 % CI	*p* value	Hazard ratio	95 % CI	*p* value
LMR: high versus low	0.337	0.129–0.543	0.001	0.239	0.236–0.593	0.001
HPV DNA status: HR-positive HPV DNA versus non-HR HPV DNA	0.243	0.036–0.535	0.001	0.267	0.054–0.772	0.006
Age: ≤46 versus >46	1.591	1.021–3.627	0.065	2.512	1.001–3.872	0.091
FIGO classification: IIA versus IIB versus IIIA versus IIIB versus IIIB versus IVA	3.486	0.957–5.248	0.035	3.275	0.845–5.328	0.073
pathological type: SCC versus ADC versus ASC versus UDC	2.523	1.093– 4.486	0.013	2.753	1.964–5.359	0.017
Lymph node status classification: positive versus negative	1.375	0.896–3.247	0.073	2.553	1.587–3.439	0.065
Treatment modality: IC + RT versus CCRT versus IC + CCRT	3. 864	3.587–6.339	0.039	2.486	1.987–4.239	0.102
ALC: high versus low	0.394	0.198–0.624	0.027	0.512	0.165–0.928	0.143
AMC: high versus low	1.703	0.138–3.614	0.181	0.982	0.301–2.124	0.713

*OS* overall survival; *PFS* progression-free survival; *CI* confidence interval; *LMR* lymphocyte-to-monocyte ratio; *HPV* human papillomavirus; *DNA* deoxyribonucleic acid; *HR* high risk; *FIGO* International Federation of Gynecology and Obstetrics; *SCC* squamous cell carcinoma; *ADC* adenocarcinoma; *ASC* adenosquamous carcinoma, *UDC* undifferentiated carcinoma; *IC* inductive chemotherapy; *CCRT* concurrent chemoradiotherapy; *RT* radiation therapy; *AMC* absolute monocyte count; *ALC* absolute lymphocyte count

For all the enrolled patients, high LMR level was confirmed to be an independent prognostic factor for superior OS (HR = 0.337, 95 % CI 0.129–0.543, *p* = 0.001) and superior PFS (HR = 0.239, 95 % CI 0.236–0.593, *p* = 0.001, Table 3).

In the concurrent study, we found that different treatment modalities had a positive impact on OS for patients with an unresectable advanced cervical carcinoma who received chemoradiation (Fig. 5b). To reduce heterogeneity of the studied cohort, we repeated the Cox analysis according to specific treatment modality. We found that LMR was still an independent prognostic factor for superior survival for each treatment group (Tables 4, 5, 6).

**Table 4:** Multivariate analysis of Cox proportional hazards model of 5-year OS and PFS for the patients of the IC + CCRT group (n = 134)

Prognostic factors	OS			PFS		
	Hazard ratio	95 % CI	*p* value	Hazard ratio	95 % CI	*p* value
LMR: high versus low	0.362	0.181–0.523	0.010	0.329	0.325–0.693	0.018
HPV DNA status: HR-positive HPV DNA versus non-HR HPV DNA	0.203	0.025–0. 425	0.001	0.317	0.104–0.672	0.028
Age: ≤46 versus >46	0.532	0.121–0.727	0.063	1.021	0.876–3.672	0.153
FIGO classification: IIA versus IIB versus IIIA versus IIIB versus IIIB versus IVA	0.186	0.087–0.439	0.035	0.238	0.0137–0.639	0.033
pathological type: SCC versus ADC versus ASC versus UDC	0.416	0.087–0.539	0.010	0.286	0.187–0.441	0.011
Lymph node status classification: positive lymph node versus negative lymph node	1.475	0.987–3.439	0.043	2.753	1.587–4.339	0.045
ALC: high versus low	0.460	0.108–0.732	0.030	0.421	0.265–0.828	0.001
AMC: high versus low	1.814	1.049–3.738	0.131	0.682	0.212–1.225	0.135

*OS* overall survival; *PFS* progression-free survival; *CI* confidence interval; *LMR* lymphocyte-to-monocyte ratio; *HPV* human papillomavirus; *DNA* deoxyribonucleic acid; *HR* high risk; *FIGO* International Federation of Gynecology and Obstetrics; *SCC* squamous cell carcinoma; *ADC* adenocarcinoma; *ASC* adenosquamous carcinoma; *UDC* undifferentiated carcinoma; *IC* inductive chemotherapy; *CCRT* concurrent chemoradiotherapy; *RT* radiation therapy; *AMC* absolute monocyte count; *ALC* absolute lymphocyte count

**Table 5:** Multivariate analysis of Cox proportional hazards model of 5-year OS and PFS for the patients of the CCRT group (n = 147)

Prognostic factors	OS			PFS		
	Hazard ratio	95 % CI	*p* value	Hazard ratio	95 % CI	*p* value
LMR: high versus low	0.251	0.161–0.412	0.013	0.218	0.014–0.582	0.021
HPV DNA status: HR-positive HPV DNA versus non-HR HPV DNA	0.132	0.023–0.314	0.001	0.206	0.064–0.516	0.017
Age: ≤46 versus >46	0.323	0.101–0.616	0.072	1.113	0.756–3.561	0.403
FIGO classification: IIA versus IIB versus IIIA versus IIIB versus IIIB versus IVA	0.174	0.074–0.338	0.024	0.127	0.017–0.528	0.023
pathological type: SCC versus ADC versus ASC versus UDC	0.305	0.056–0.428	0.001	0.275	0.126–0.490	0.031
Lymph node status classification: positive lymph node versus negative lymph node	1.364	0.789–3.238	0.063	2.531	1.476–4.821	0.059
ALC: high versus low	0.458	0.198–0.824	0.021	0.512	0.165–0.928	0.013
AMC: high versus low	0.703	0.149–1.614	0.219	0.982	0.301–2.124	0.533

*OS* overall survival; *PFS* progression-free survival; *CI* confidence interval; *LMR* lymphocyte-to-monocyte ratio; *HPV* human papillomavirus; *DNA* deoxyribonucleic acid; *HR* high risk; *FIGO* International Federation of Gynecology and Obstetrics; *SCC* squamous cell carcinoma; *ADC* adenocarcinoma; *ASC* adenosquamous carcinoma; *UDC* undifferentiated carcinoma; *IC* inductive chemotherapy; *CCRT* concurrent chemoradiotherapy; *RT* radiation therapy; *AMC* absolute monocyte count; *ALC* absolute lymphocyte count

**Table 6:** Multivariate analysis of Cox proportional hazards model of 5-year OS and PFS for the patients of the IC + RT group (n = 143)

Prognostic factors	OS			PFS		
	Hazard ratio	95 % CI	*p* value	Hazard ratio	95 % CI	*p* value
LMR: high versus low	0.471	0.229–0.634	0.010	0.430	0.436–0.794	0.027
HPV DNA status: HR-positive HPV DNA versus non-HR HPV DNA	0.314	0.036–0.536	0.012	0.418	0.215–0.738	0.039
Age: ≤46 versus >46	0.643	0.232–0.837	0.074	1.132	0.987–3.783	0.264
FIGO classification: IIA versus IIB versus IIIA versus IIIB versus IIIB versus IVA	0.297	0.198–0.540	0.045	0.395	0.0245–0.740	0.044
pathological type: SCC versus ADC versus ASC versus UDC	0.527	0.186–0.640	0.036	0.397	0.298–0.552	0.023
Lymph node status classification: positive lymph node versus negative lymph node	2.586	0.987–4.540	0.073	2.753	1.747–5.394	0.065
ALC: high versus low	0.548	0.098–0.931	0.012	0.589	0.065–0.938	0.015
AMC: high versus low	0.829	0.152–1.730	0.321	0.771	0.413–2.313	0.421

*OS* overall survival; *PFS* progression-free survival; *CI* confidence interval; *LMR* lymphocyte-to-monocyte ratio; *HPV* human papillomavirus; *DNA* deoxyribonucleic acid; *HR* high risk; *FIGO* International Federation of Gynecology and Obstetrics; *SCC* squamous cell carcinoma; *ADC* adenocarcinoma; *ASC* adenosquamous carcinoma; *UDC* undifferentiated carcinoma; *IC* inductive chemotherapy; *CCRT* concurrent chemoradiotherapy; *RT* radiation therapy; *AMC* absolute monocyte count; *ALC* absolute lymphocyte count

In the multivariate analysis, among the three combined modality as mentioned above, the low LMR and concurrent non-HR HPV DNA status were the negative prognostic factors for OS (HR = 2.970, 95 % CI 2.075–5.141, *p* = 0.000) and PFS (HR = 1.844, 95 % CI 1.348–2.523, *p* = 0.000), respectively.

## Discussion

To the best of our knowledge, this study is the first to explore the positive prognostic effect of high LMR on survival and its close association with HPV DNA status of pretreatment cancer tissue in patients with unresectable advanced cervical carcinoma treated with chemoradiotherapy. Elevated level of pretreatment ALC and HR-positive HPV DNA status were provide to be significantly correlated with superior OS and PFS. LMR and HPV DNA status were independent prognostic factors, and their combined detection can be of better prognostic value for OS and PFS, which can be used to stratify patients with unresectable advanced cervical carcinoma at HR of unfavorable prognosis.

In the last decade, pretreatment peripheral differential leukocytes (including neutrophil, lymphocyte, and monocyte) have been observed to be associated with prognosis of various cancers. Survival outcomes in cervical carcinoma patients are influenced by immune cells [17, 18] in the tumor microenvironment, including tumor-infiltrating lymphocytes and neutrophils. However, in clinical settings, the prognostic value of the LMR and monocytes as well as its correlation with HR-positive HPV DNA status have not been elucidated in cervical cancer but have been observed in other cancers [19–30].

The prognostic influence of lymphocyte and monocyte, as the major components of inflammatory response, have been extensively investigated on cancer patients. In the current study, the increased circulating lymphocyte count (≥2.35 × 10^9^/L) was closely correlated with better OS in unresectable advanced cervical carcinoma under univariate and multivariate analysis. This result is consistent with the study of Choi et al. who reported that high lymphocyte count (≥2.145 × 10^9^/L) remains an independent factor for favorable survival in patients with unresectable advanced cervical carcinoma [31]. However, ALC failed to maintain its prognostic role in some other tumors [24, 32]. No clear data are currently available to explain this problem. A possible explanation could be the different cutoff values of the absolute lymphocyte count observed by different studies. Another possible explanation is that different immune subtypes of lymphocytes can have different influences on biological effects and prognosis in different tumors, apart from the pure quantity. For example, tumor-infiltrating lymphocytes (TILs) establish a defense barrier against cancer, whereas regulatory T-cells may have inhibitory effects on antitumor immunity. Furthermore, some studies showed that higher levels of TILs, including CD8+ T-cells in some cancers, are correlated with a favorable survival [33], whereas regulatory T cell infiltration in pancreatic carcinoma has been found to be an unfavorable prognostic factor [34, 35].

Monocytes, as either inflammatory or immune cells, can be mobilized from the bone marrow to the peripheral blood and are furtherly prompted into tissue macrophages in the inflammation setting [36]. On this basis, macrophages in the tumor microenvironment can accelerate cancer-related angiogenesis, invasiveness, immunosuppression, and cell seeding metastasis [37, 38]. Patients with cervical cancer can be divided into superior- and inferior-prognosis subgroups based on pretreatment circulating AMC [39]. These previous studies indicated that peripheral blood monocytes may be linked to unfavorable prognosis. However, unlike the previous studies, the present study failed to reveal that pretreatment peripheral blood AMC showed significant correlation with long-time treatment outcome in the setting of unresectable advanced cervical carcinoma. However, patients with HR-positive HPV DNA have higher monocyte counts than those with negative HPV DNA. Although various mechanisms have yet be confirmed through studies, we speculate that higher monocyte counts may be caused by the activation and release of virus-derived proinflammatory cytokines, such as GM-CSF, TNF-alpha and IL-12, in the inflammation setting.

Pretreatment LMR serves as an independent prognostic factor for survival in some malignancies excluding cervical carcinoma [7, 9, 24]. However, the prognostic significance of LMR in cervical cancer has not been clarified. The present study is the first to find a positive effect of high LMR on survival in unresectable advanced cervical carcinoma. In particular, patients in the high LMR group had a significantly longer OS than those in the low LMR group (25.0 versus 16.0 months, *p* < 0.001), for all the enrolled patients. Moreover, high LMR level was confirmed to be an independent prognostic factor for superior survival (HR = 0.337, 95 % CI 0.129–0.543, *p* < 0.01, Table 3). As is known to us, the different treatment modality may have some effect on treatment outcome. Similarly, we also found that different treatment modality had a positive impact on OS for patients with an unresectable advanced cervical carcinoma who received chemoradiation (Fig. 5). To reduce heterogeneity of the studied cohort, we repeated the Cox analysis according to specific treatment modality. We found that LMR was an independent prognostic factor for superior survival for each treatment group (Tables 4, 5, 6). In the current study, we only discussed the favorable prognostic impact of high LMR level for patients with an unresectable advanced cervical carcinoma. For cervical cancer patients who undergo a radical operation, a newer study also demonstrated that a decreased pretreatment LMR is associated with a poor prognosis [40] patients with stage Ib1-IIa cervical cancer. Based on these facts, we could conclude that pretreatment high LMR is associated with a good prognosis in cervical carcinoma.

In the current study, we found that the similarity of clinical implication for these two parameters (pretreatment ALC and LMR) did exist. For example, firstly, elevated level of pretreatment ALC and LMR were provided to be significantly correlated with superior OS and PFS under an univariate analysis (Table 2); Secondly, like pretreatment LMR, ALC was also an independent prognostic factor for OS under multivariate analysis for all the enrolled patients (Table 3) or those from each treatment group (Tables 4, 5, 6); Thirdly, both ALC and LMR could be used to stratify patients at HR of malignancy-related death (Figs. 2, 3a, b). However, the differences of clinical implication for these two parameters were also observed. Firstly, the former had sensitivity of 77.73 % and specificity of 72.4 % as a predictor of OS, whereas the latter had sensitivity of 85.37 % and specificity of 75.40 % (Fig. 1a, c). Secondly, previous study demonstrated that several conditions, such as chronic inflammatory diseases, active infection, and smoking, may influence the specificity of both markers [41], although ALC and AMC are easy to measure. LMR superiority is due to the stability of the ratio compared with the absolute cell counts [42]. Based on the reasons mentioned above, we concluded that LMR is more accurate than ALC as a predictor in cervical carcinoma.

Previous studies have demonstrated that the hyperactivation of the inflammatory pathways subsequent to viral infection is a driving force that accelerates cancer development, malignant traits, and progression of cervical cancer [43, 44]. Moreover, previous data demonstrated that negative HPV is linked to poor prognosis in invasive cervical cancer [11]. Similar with most previous results, this study also found that patients in the HR-positive HPV DNA group showed higher 5-year OS and PFS rates than those in the non-HR HPV DNA group. Moreover, HPV DNA status was an independent prognostic factor for PFS and OS (Table 2).

We classified the enrolled patients according to the detected HPV DNA status from pretreatment cancer tissue, in order to elucidate the influence of HPV DNA status on the prognosis of LMR level in cervical cancer. We observed that higher LMR level was an independent prognostic factor for superior survival in the HR-positive HPV DNA group. In the non-HR HPV DNA group, the findings were contrary to those in the HR-positive HPV DNA group. No significant difference on PFS and OS was found between the two groups, thereby supporting the concept that the prognostic effect of LMR in advanced cervical carcinoma may be correlated with the HPV DNA status.

We further investigated the clinical value of joint detection of pretreatment LMR level and HPV DNA status in a single patient. Patients with high LMR and concurrent HR-positive HPV DNA showed the best prognostic factor because of their longer survival compared with other groups on OS and PFS (*p* < 0.01, both). Moreover, high LMR and concurrent HR-positive HPV DNA were found to be independent predictive markers of OS.

This study has several limitations given its retrospective nature. First, selection bias cannot be completely eliminated in the study cohort. Therefore, caution should be taken while interpreting the results of this study. Second, during stratified analysis, the sample size was too small to provide sufficiently persuasive evidence to support any given conclusion. Based on these limitations, a prospective study should be conducted to provide more evidence to support the conclusions of this study.

## Conclusions

This study is the first to demonstrate that pretreatment elevated peripheral blood LMR can predict favorable prognosis and is an independent prognostic factor for OS and PFS in locally advanced cervical cancer, along with HR-HPV DNA status. The different effects of LMR in combined chemoradiotherapy on survival may be related to the HR-HPV DNA status of pretreatment cancer tissue in patients with locally advanced cervical carcinoma. The combined detection of LMR and HR-HPV DNA status can contribute in the correct stratification of patients with unresectable advanced cervical carcinoma at HR of unfavorable prognosis. We acknowledge that this finding is limited to a retrospective study in a single center. Thus, further studies are necessary to be performed in a multicenter or prospective manner to validate these important findings.

## Abbreviations

OS: overall survival
PFS: progression-free survival
CI: confidence interval
LMR: lymphocyte-to-monocyte ratio
HPV: human papillomavirus
DNA: deoxyribonucleic acid
HR: high risk
FIGO: International Federation of Gynecology and Obstetrics
SCC: squamous cell carcinoma
ADC: adenocarcinoma
ASC: adenosquamous carcinoma
UDC: undifferentiated carcinoma
IC: inductive chemotherapy
CCRT: concurrent chemoradiotherapy
RT: radiation therapy
AMC: absolute monocyte count
ALC: absolute lymphocyte
